# Crystal structure of 1-[(2,3-dihydro-1*H*-naphtho­[1,2-*e*][1,3]oxazin-2-yl)meth­yl]naphthalen-2-ol: a possible candidate for new polynaphthoxazine materials

**DOI:** 10.1107/S2056989015015583

**Published:** 2015-08-26

**Authors:** Augusto Rivera, Jicli José Rojas, Jaime Ríos-Motta, Michael Bolte

**Affiliations:** a Universidad Nacional de Colombia, Sede Bogotá, Facultad de Ciencias, Departamento de Química, Cra 30 No. 45-03, Bogotá, Código Postal 111321, Colombia; b Institut für Anorganische Chemie, J. W. Goethe-Universität Frankfurt, Max-von Laue-Str. 7, 60438 Frankfurt/Main, Germany

**Keywords:** crystal structure, polynaphthoxazine materials, oxazine, intra­molecular hydrogen bond, C—H⋯π inter­actions, π–π inter­action

## Abstract

In the title compound, C_23_H_19_NO_2_, an oxazine Mannich base derivative, the oxazine ring has a half-chair conformation. The 2-hy­droxy­naphthalen-1-yl substituent is placed in an axial position. There is an intra­molecular O—H⋯N hydrogen bond, forming an *S*(6) graph-set motif. In the crystal, mol­ecules are connected by a pair of C—H⋯π inter­actions into an inversion dimer, which is reinforced by another pair of weak C—H⋯π inter­actions. The dimers are linked by a π–π inter­action [centroid-centroid distance = 3.6268 (17) Å], consolidating a column along the *a* axis. Furthermore, the columns inter­act with each other by a weak C—H⋯π inter­action, generating a three-dimensional network.

## Abstract 2

A novel naphthoxazine has been synthesized and structurally characterized. In the crystal, pairs of inversion-related mol­ecules are linked into inversion dimers *via* C—H⋯π inter­actions.

## Chemical context   

Benzoxazines and naphthoxazines have been shown to polymerize *via* a thermally induced ring-opening reaction of the oxazine ring to form a phenolic structure associated with traditional phenolic resins (Ishida & Sanders, 2001 ▸). Polybenzoxazines, polynaphthoxazines and their derivatives are a class of phenolic resins which are alternative to the traditional resins (Yildirim *et al.*, 2006 ▸). So far the main contribution to the chemistry of these compounds has been the work of Burke (Burke, 1949 ▸; Burke *et al.*, 1952 ▸), who was the first to show that aromatic oxazines could be obtained *via* Mannich-type condensation–cyclization reactions of certain phenols or naphtols with formaldehyde and primary amines in the molar ratio of 1:2:1. Various methods have been reported for the synthesis of di­hydro-1,3-oxazines including the reaction under neat conditions *via* Mannich-type condensation–cyclization reaction of phenols or naphthols with formaldehyde and primary amines (Mathew *et al.*, 2010 ▸). Our current research includes synthesis and characterization of monofunctional benzoxazines using aminals as performed Mannich electrophiles instead of formaldehyde and primary amines. Earlier (Rivera *et al.*, 2005 ▸), we have reported an inter­esting behaviour of the macrocyclic aminal 1,3,6,8-tetra­aza­tri­cyclo[4.4.1.1^3,8^]dodecane (TATD) with hindered *meta*-disubstituted phenols affording 3,3-ethyl­ene-bis­(3,4-di­hydro-2*H*-1,3-benzo­xazines) with good yields by a Mannich-type reaction in basic media. Recently, we synthesized the title compound by a reaction between the cyclic aminal 1,3,6,8-tetra­aza­tri­cyclo­[4.3.1.1.^3,8^]undecane (TATU) with 2-naphthol solvent-free at low temperature. Because a wide range of cured properties can be obtained (Uyar *et al.*, 2008 ▸) depending on the structure of aryl­oxazine monomers, initiators and the curing conditions, the title compound is a very good candidate as a monomer for the investigation of the polymerization of this class of compounds.
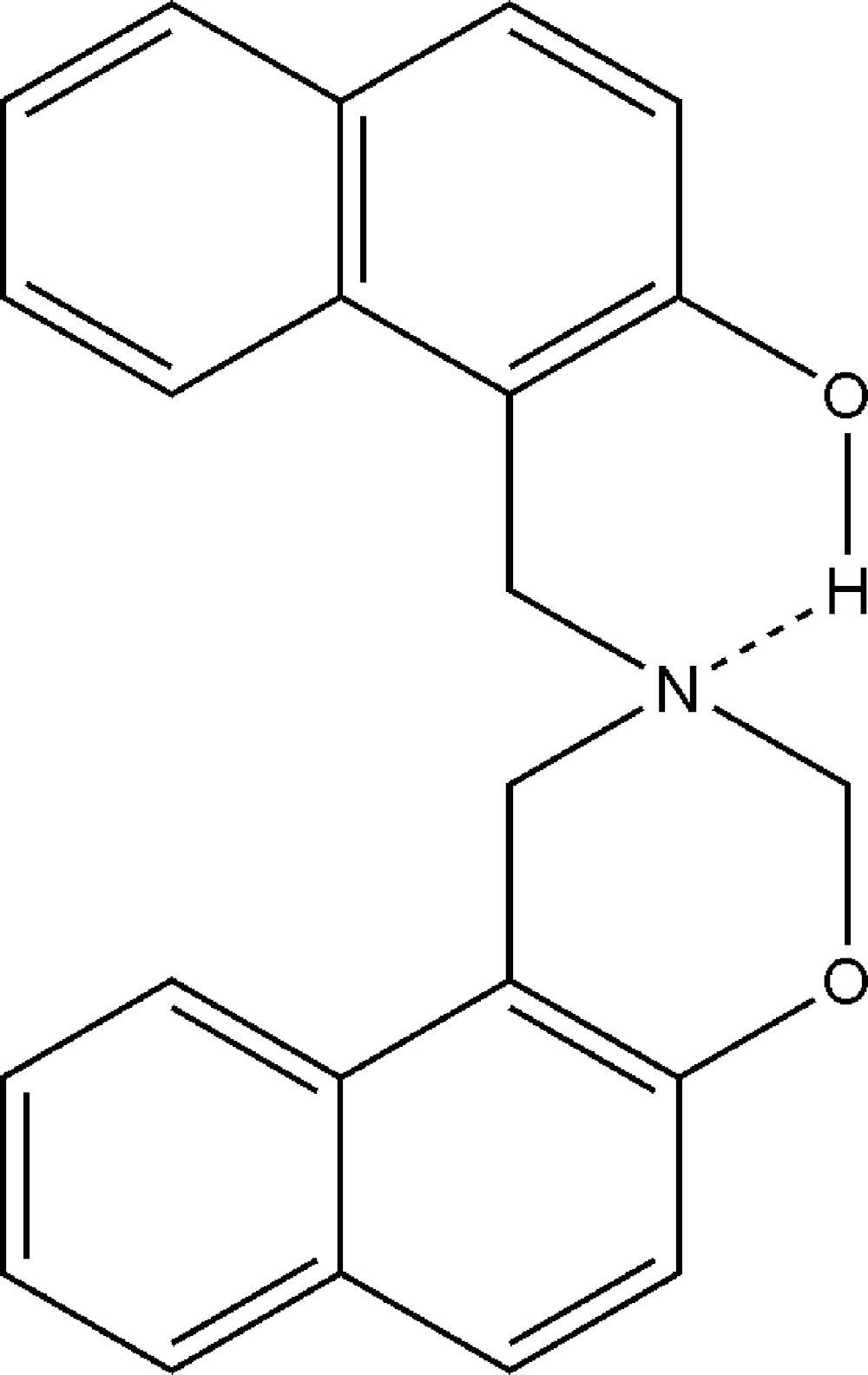


## Structural commentary   

**Figure 1:**
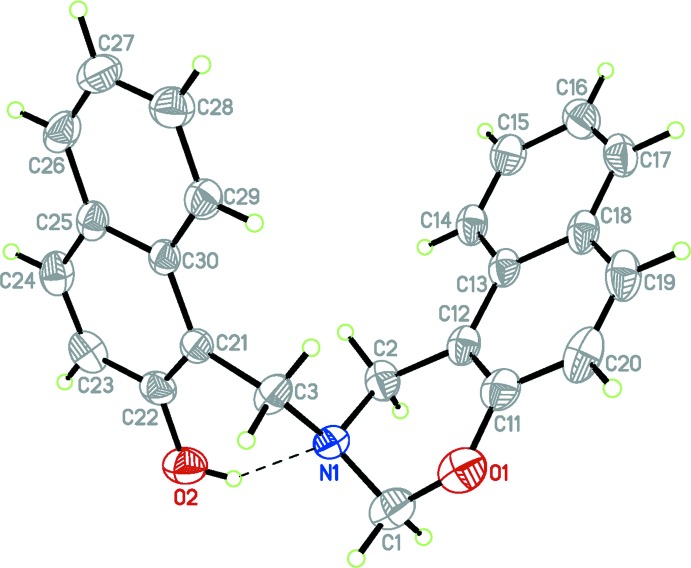
The mol­ecular structure of the title compound, Displacement ellipsoids are drawn at the 50% probability level. The hydrogen bond is shown as a dashed line.

The mol­ecular structure of the title compound is shown in Fig. 1 ▸. The six-membered oxazine ring adopts a half-chair conformation with atoms N1 and C1 displaced by 0.323 (2) and 0.292 (3) Å, respectively, from the mean plane composed of atoms O1, C11, C12 and C2. The puckering parameters are *Q* = 0.479 (3) Å, *θ* = 50.0 (3)° and *φ* = 98.3 (4)° for the ring O1/C1/N1/C2/C12/C11. The (2-hy­droxy­naphthalen-1-yl)methyl group bonded to atom N1 of the oxazine ring is placed in an axial position. The pendant naphthyl group (C21–C30) makes a dihedral angle of 59.94 (4)° with the oxazine ring plane defined by atoms C11, C12 and O1. The bond lengths, N1—C1 and O1—C1, are normal and comparable to the corresponding values observed in the related structure of 6-bromo-2,4-bis­(3-meth­oxy-phen­yl)-3,4-di­hydro-2*H*-1,3-naphthoxazine (Sarojini *et al.*, 2007 ▸). There is an intra­molecular O—H⋯N hydrogen bond (Table 1 ▸), forming an *S*(6) graph-set motif, where the N⋯O distance is longer by about 0.04 and 0.03 Å, respectively, than the observed values in related structures of 1-(piperidin-1-ylmeth­yl)-2-naphthol (Liu *et al.*, 2005 ▸) and 1-morpholino­methyl-2-naphthol (Ma *et al.*, 2005 ▸).

## Supra­molecular features   

**Figure 2:**
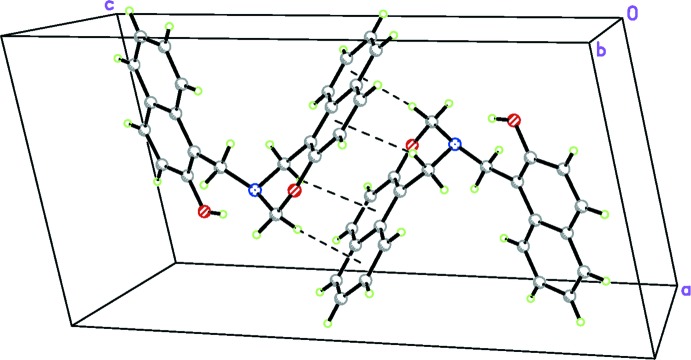
An inversion dimer in the crystal of the title compound, with C—H⋯π inter­actions indicated by dashed lines.

**Figure 3:**
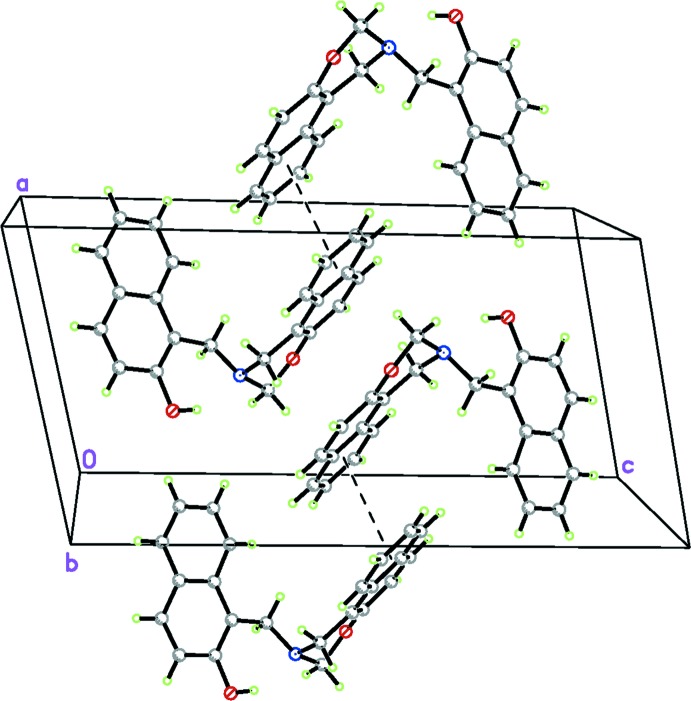
The view of the column structure along the *a* axis, showing the π–π stacking inter­actions (dashed lines).

**Figure 4:**
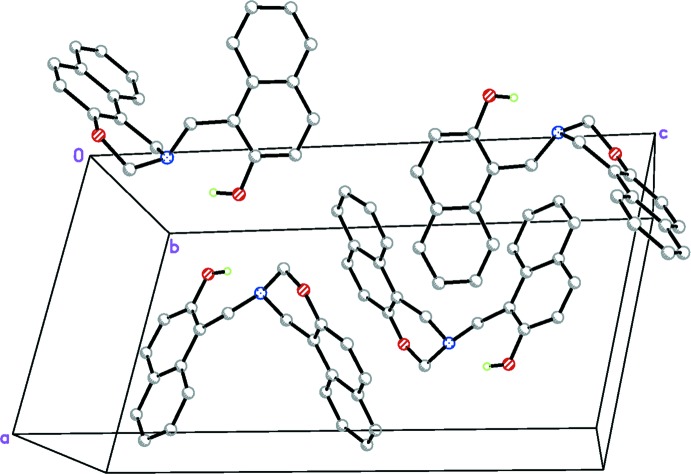
Packing diagram of the title compound. C-bound H atoms have been omitted for clarity.

The crystal packing organization is essentially the result of two different types of inter­actions involving inversion-related mol­ecules. Based on the distance criteria employed in *PLATON* (Spek, 2009 ▸), the most notable inter­molecular contact is a C—H⋯π inter­action (C1—H1*A*⋯*Cg*3^i^; Table 1 ▸), so that an inversion dimer is formed (Fig. 2 ▸). In addition, there is another C—H⋯π inter­action (C2—H2*B*⋯*Cg*2^i^; Table 1 ▸) in the dimer. A column of alternating inversion dimers extending along the *a* axis results from a π–π stacking inter­action (Fig. 3 ▸) between adjacent 2,3-di­hydro-1*H*-naphtho­[1,2-*e*][1,3]oxazine ring systems with a centroid–centroid distance of 3.6268 (17) Å [*Cg*3⋯*Cg*3^iii^; symmetry code: (iii) = −*x*, −*y* + 1, −*z* + 1]. Neighboring columns are connected by a weak C—H⋯π inter­action (C14—H14*A*⋯*Cg*5^ii^; Table 1 ▸), generating a three-dimensional network. The unit-cell packing is shown in Fig. 4 ▸.

## Database survey   

The 2,3-di­hydro-1*H*-naphtho­[1,2-*e*][1,3]oxazine fragment is a quite rigid moiety. A search in the CSD (Groom & Allen, 2014 ▸) for this fragment gave 22 hits with 24 fragments. The torsion angles in the heterocycle show broadly consistent values. Their absolute values are in the following ranges: O—C—N—C 56.0–69.7°, C—N—C—C 37.7–53.8°, N—C—C—C 3.7–24.2°, C—C—C—O 0.1–6.3°, C—C—O—C 1.0–21.6° and C—O—C—N 28.8–56.1°. Thus, it can be concluded that the conformation of this heterocycle is the same in all fragments. The values of the title compound fit very well into these ranges: O1—C1—N1—C2 64.2 (3)°, C1—N1—C2—C12 − 48.9 (3)°, N1—C2—C12—C11 19.3 (3)°, C2—C12—C11—O1 − 0.5 (3)°, C12—C11—O1—C1 12.4 (3)° and C11—O1—C1—N1 − 45.3 (3)°.

## Synthesis and crystallization   

2-Naphthol (144 mg, 1 mmol) and 1,3,6,8-tetra­aza­tri­cyclo­[4.3.1.1^3,8^]undecane (TATU) (154 mg, 1 mmol) were manually ground together, heated to 313 K and stirred for 12 h under solvent-free conditions. Progress of the reaction was determined by TLC monitoring. After completion of the reaction, the mixture was cooled to room temperature and the solid residue was purified by silica gel column chromatography with benzene–ethyl acetate (4:1) as the eluent to give 1-{[1*H*-naphtho­[1,2-*e*][1,3]oxazin-2(3*H*)-yl]meth­yl}naphthalen-2-ol as a brown solid in 28% yield. This compound was obtained in its crystalline form by recrystallization from an absolute ethanol solution (m.p. 443 K).

## Refinement   

Crystal data, data collection and structure refinement details are summarized in Table 2 ▸. All H atoms were located in a difference electron-density map. The hydroxyl H atom was refined using a riding-model approximation with O—H = 0.84 Å. The *U*_iso_(H) value and the C—C—O—H torsion angle were refined. C-bound H atoms were fixed geometrically (C—H = 0.95 or 0.99 Å) and treated as riding with *U*_iso_(H) = 1.2*U*_eq_(C).

## Supplementary Material

Crystal structure: contains datablock(s) I, global. DOI: 10.1107/S2056989015015583/is5412sup1.cif

Structure factors: contains datablock(s) I. DOI: 10.1107/S2056989015015583/is5412Isup2.hkl

Supporting information file. DOI: 10.1107/S2056989015015583/is5412Isup3.cml

CCDC reference: 1419687

Additional supporting information:  crystallographic information; 3D view; checkCIF report

## Figures and Tables

**Table 1:** Hydrogen-bond geometry (, ) *Cg*2, *Cg*3 and *Cg*5 are the centroids of the C11C13/C18C20, C13C18 and C25C30 rings, respectively.

*D*H*A*	*D*H	H*A*	*D* *A*	*D*H*A*
O2H2N1	0.84	1.88	2.627(2)	147
C1H1*A* *Cg*3^i^	0.99	2.53	3.501(3)	169
C2H2*B* *Cg*2^i^	0.99	2.86	3.743(3)	149
C14H14*Cg*5^ii^	0.99	2.87	3.723(3)	150

Symmetry codes: (i) 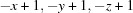; (ii) 
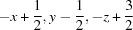
.

**Table 2:** Experimental details

Crystal data	
Chemical formula	C_23_H_19_NO_2_
*M*_r_	341.39
Crystal system, space group	Monoclinic, *P*2_1_/*n*
Temperature (K)	173
*a*, *b*, *c* ()	9.6570(12), 9.7609(7), 18.790(2)
()	102.331(10)
*V* (^3^)	1730.3(3)
*Z*	4
Radiation type	Mo *K*
(mm^1^)	0.08
Crystal size (mm)	0.31 0.11 0.11

Data collection	
Diffractometer	STOE IPDS II two-circle
Absorption correction	Multi-scan (*X-AREA*; Stoe Cie, 2001 ▸)
*T*_min_, *T*_max_	0.300, 0.991
No. of measured, independent and observed [*I* > 2(*I*)] reflections	8807, 3222, 2069
*R*_int_	0.051
(sin /)_max_ (^1^)	0.608

Refinement	
*R*[*F* ^2^ > 2(*F* ^2^)], *wR*(*F* ^2^), *S*	0.053, 0.135, 0.96
No. of reflections	3222
No. of parameters	237
H-atom treatment	H-atom parameters constrained
_max_, _min_ (e ^3^)	0.57, 0.24

Computer programs: *X-AREA* (Stoe Cie, 2001 ▸), *SHELXS97*, *XP* in *SHELXTL-Plus* and *SHELXL97* (Sheldrick, 2008 ▸), *SHELXL2014*/6 (Sheldrick, 2015 ▸).

## supplementary crystallographic information

### Crystal data

**Table:**

C_23_H_19_NO_2_	*F*(000) = 720
*M**_r_* = 341.39	*D*_x_ = 1.310 Mg m^−^^3^
Monoclinic, *P*2_1_/*n*	Mo *K*α radiation, λ = 0.71073 Å
*a* = 9.6570 (12) Å	Cell parameters from 5838 reflections
*b* = 9.7609 (7) Å	θ = 3.4–25.8°
*c* = 18.790 (2) Å	µ = 0.08 mm^−^^1^
β = 102.331 (10)°	*T* = 173 K
*V* = 1730.3 (3) Å^3^	Needle, colourless
*Z* = 4	0.31 × 0.11 × 0.11 mm

### Data collection

**Table:**

STOE IPDS II two-circle-diffractometer	2069 reflections with *I* > 2σ(*I*)
Radiation source: Genix 3D IµS microfocus X-ray source	*R*_int_ = 0.051
ω scans	θ_max_ = 25.6°, θ_min_ = 3.4°
Absorption correction: multi-scan (*X-AREA*; Stoe & Cie, 2001)	*h* = −9→11
*T*_min_ = 0.300, *T*_max_ = 0.991	*k* = −10→11
8807 measured reflections	*l* = −22→22
3222 independent reflections	

### Refinement

**Table:**

Refinement on *F*^2^	0 restraints
Least-squares matrix: full	Hydrogen site location: inferred from neighbouring sites
*R*[*F*^2^ > 2σ(*F*^2^)] = 0.053	H-atom parameters constrained
*wR*(*F*^2^) = 0.135	*w* = 1/[σ^2^(*F*_o_^2^) + (0.0738*P*)^2^] where *P* = (*F*_o_^2^ + 2*F*_c_^2^)/3
*S* = 0.96	(Δ/σ)_max_ < 0.001
3222 reflections	Δρ_max_ = 0.57 e Å^−^^3^
237 parameters	Δρ_min_ = −0.24 e Å^−^^3^

### Special details

**Table:**

Geometry. All e.s.d.'s (except the e.s.d. in the dihedral angle between two l.s. planes) are estimated using the full covariance matrix. The cell e.s.d.'s are taken into account individually in the estimation of e.s.d.'s in distances, angles and torsion angles; correlations between e.s.d.'s in cell parameters are only used when they are defined by crystal symmetry. An approximate (isotropic) treatment of cell e.s.d.'s is used for estimating e.s.d.'s involving l.s. planes.

### Fractional atomic coordinates and isotropic or equivalent isotropic displacement parameters (Å^2^)

**Table:**

	*x*	*y*	*z*	*U*_iso_*/*U*_eq_	
O1	0.5357 (2)	0.80192 (19)	0.57257 (9)	0.0422 (5)	
O2	0.67542 (18)	0.5355 (2)	0.80171 (9)	0.0428 (5)	
H2	0.6763	0.5678	0.7604	0.039 (8)*	
N1	0.5689 (2)	0.6438 (2)	0.67377 (9)	0.0332 (5)	
C1	0.6360 (3)	0.7296 (3)	0.62925 (13)	0.0427 (6)	
H1A	0.6988	0.6725	0.6060	0.051*	
H1B	0.6961	0.7976	0.6607	0.051*	
C2	0.4914 (3)	0.5345 (3)	0.62777 (10)	0.0336 (6)	
H2A	0.4305	0.4846	0.6553	0.040*	
H2B	0.5598	0.4684	0.6149	0.040*	
C3	0.4775 (3)	0.7190 (3)	0.71422 (11)	0.0326 (5)	
H3A	0.3892	0.7470	0.6801	0.039*	
H3B	0.5269	0.8031	0.7356	0.039*	
C11	0.4279 (3)	0.7211 (3)	0.53568 (11)	0.0346 (6)	
C12	0.4002 (2)	0.5935 (2)	0.55868 (10)	0.0293 (5)	
C13	0.2837 (2)	0.5172 (3)	0.51787 (10)	0.0292 (5)	
C14	0.2464 (3)	0.3851 (3)	0.53810 (10)	0.0328 (6)	
H14	0.3030	0.3424	0.5798	0.039*	
C15	0.1310 (3)	0.3174 (3)	0.49924 (11)	0.0381 (6)	
H15	0.1076	0.2294	0.5148	0.046*	
C16	0.0471 (3)	0.3762 (3)	0.43679 (11)	0.0407 (6)	
H16	−0.0330	0.3284	0.4100	0.049*	
C17	0.0809 (3)	0.5027 (3)	0.41453 (11)	0.0388 (7)	
H17	0.0246	0.5414	0.3716	0.047*	
C18	0.1980 (3)	0.5774 (3)	0.45412 (10)	0.0336 (6)	
C19	0.2309 (3)	0.7106 (3)	0.43329 (11)	0.0384 (6)	
H19	0.1742	0.7510	0.3909	0.046*	
C20	0.3419 (3)	0.7818 (3)	0.47263 (11)	0.0418 (7)	
H20	0.3620	0.8716	0.4582	0.050*	
C21	0.4405 (2)	0.6320 (2)	0.77453 (10)	0.0272 (5)	
C22	0.5430 (2)	0.5495 (3)	0.81558 (11)	0.0311 (5)	
C23	0.5180 (3)	0.4759 (3)	0.87625 (11)	0.0370 (6)	
H23	0.5917	0.4229	0.9050	0.044*	
C24	0.3891 (3)	0.4808 (3)	0.89361 (11)	0.0360 (6)	
H24	0.3736	0.4314	0.9348	0.043*	
C25	0.2767 (3)	0.5584 (2)	0.85145 (10)	0.0298 (5)	
C26	0.1401 (3)	0.5588 (3)	0.86679 (11)	0.0365 (6)	
H26	0.1231	0.5070	0.9069	0.044*	
C27	0.0318 (3)	0.6318 (3)	0.82539 (12)	0.0406 (6)	
H27	−0.0603	0.6286	0.8357	0.049*	
C28	0.0574 (3)	0.7115 (3)	0.76759 (12)	0.0392 (6)	
H28	−0.0175	0.7637	0.7392	0.047*	
C29	0.1886 (2)	0.7151 (3)	0.75153 (10)	0.0326 (6)	
H29	0.2037	0.7713	0.7126	0.039*	
C30	0.3031 (2)	0.6371 (2)	0.79162 (9)	0.0257 (5)	

### Atomic displacement parameters (Å^2^)

**Table:**

	*U*^11^	*U*^22^	*U*^33^	*U*^12^	*U*^13^	*U*^23^
O1	0.0455 (11)	0.0355 (11)	0.0494 (9)	−0.0013 (9)	0.0184 (8)	−0.0046 (7)
O2	0.0317 (9)	0.0460 (12)	0.0510 (10)	0.0000 (9)	0.0094 (7)	−0.0033 (8)
N1	0.0275 (10)	0.0433 (13)	0.0306 (8)	−0.0053 (10)	0.0104 (7)	−0.0037 (8)
C1	0.0414 (15)	0.0423 (17)	0.0485 (13)	−0.0026 (14)	0.0192 (11)	−0.0015 (11)
C2	0.0348 (13)	0.0365 (15)	0.0293 (10)	0.0095 (12)	0.0061 (9)	−0.0027 (9)
C3	0.0407 (14)	0.0279 (13)	0.0334 (10)	−0.0053 (12)	0.0176 (9)	−0.0032 (9)
C11	0.0358 (13)	0.0375 (15)	0.0345 (10)	0.0053 (13)	0.0164 (9)	−0.0074 (10)
C12	0.0332 (13)	0.0302 (14)	0.0269 (9)	0.0099 (11)	0.0117 (9)	0.0005 (8)
C13	0.0315 (12)	0.0341 (14)	0.0238 (9)	0.0110 (11)	0.0102 (8)	−0.0004 (8)
C14	0.0400 (14)	0.0332 (14)	0.0263 (9)	0.0112 (12)	0.0095 (9)	0.0018 (9)
C15	0.0448 (15)	0.0362 (15)	0.0349 (11)	0.0028 (13)	0.0123 (10)	−0.0023 (10)
C16	0.0379 (14)	0.0501 (18)	0.0335 (11)	0.0070 (14)	0.0064 (10)	−0.0089 (11)
C17	0.0374 (14)	0.0524 (19)	0.0260 (10)	0.0163 (13)	0.0052 (9)	−0.0009 (10)
C18	0.0387 (14)	0.0398 (15)	0.0246 (9)	0.0166 (12)	0.0118 (9)	0.0034 (9)
C19	0.0486 (15)	0.0387 (15)	0.0283 (10)	0.0165 (14)	0.0092 (10)	0.0070 (10)
C20	0.0623 (18)	0.0313 (15)	0.0382 (11)	0.0129 (14)	0.0252 (11)	0.0062 (10)
C21	0.0310 (12)	0.0256 (12)	0.0258 (9)	−0.0078 (11)	0.0077 (8)	−0.0066 (8)
C22	0.0284 (12)	0.0302 (14)	0.0341 (10)	−0.0033 (11)	0.0052 (9)	−0.0087 (9)
C23	0.0418 (15)	0.0288 (14)	0.0360 (11)	0.0030 (12)	−0.0014 (10)	−0.0002 (9)
C24	0.0515 (16)	0.0279 (13)	0.0282 (10)	−0.0035 (13)	0.0077 (10)	0.0029 (9)
C25	0.0405 (14)	0.0245 (13)	0.0257 (9)	−0.0071 (11)	0.0099 (9)	−0.0045 (8)
C26	0.0453 (15)	0.0343 (15)	0.0349 (11)	−0.0123 (13)	0.0193 (10)	−0.0028 (10)
C27	0.0354 (14)	0.0432 (17)	0.0475 (12)	−0.0084 (13)	0.0188 (10)	−0.0115 (11)
C28	0.0335 (13)	0.0420 (16)	0.0416 (11)	0.0011 (13)	0.0070 (10)	−0.0017 (11)
C29	0.0349 (13)	0.0338 (14)	0.0293 (10)	−0.0022 (12)	0.0075 (9)	0.0029 (9)
C30	0.0308 (12)	0.0239 (12)	0.0228 (8)	−0.0061 (10)	0.0064 (8)	−0.0038 (8)

### Geometric parameters (Å, º)

**Table:**

O1—C11	1.370 (3)	C16—H16	0.9500
O1—C1	1.460 (3)	C17—C18	1.416 (4)
O2—C22	1.366 (3)	C17—H17	0.9500
O2—H2	0.8400	C18—C19	1.413 (4)
N1—C1	1.433 (3)	C19—C20	1.357 (4)
N1—C2	1.473 (3)	C19—H19	0.9500
N1—C3	1.477 (3)	C20—H20	0.9500
C1—H1A	0.9900	C21—C22	1.377 (3)
C1—H1B	0.9900	C21—C30	1.431 (3)
C2—C12	1.518 (3)	C22—C23	1.411 (4)
C2—H2A	0.9900	C23—C24	1.353 (4)
C2—H2B	0.9900	C23—H23	0.9500
C3—C21	1.518 (3)	C24—C25	1.418 (3)
C3—H3A	0.9900	C24—H24	0.9500
C3—H3B	0.9900	C25—C26	1.409 (3)
C11—C12	1.363 (4)	C25—C30	1.429 (3)
C11—C20	1.423 (3)	C26—C27	1.364 (4)
C12—C13	1.429 (3)	C26—H26	0.9500
C13—C14	1.412 (4)	C27—C28	1.400 (4)
C13—C18	1.429 (3)	C27—H27	0.9500
C14—C15	1.365 (4)	C28—C29	1.364 (3)
C14—H14	0.9500	C28—H28	0.9500
C15—C16	1.399 (3)	C29—C30	1.420 (3)
C15—H15	0.9500	C29—H29	0.9500
C16—C17	1.365 (4)		
			
C11—O1—C1	113.9 (2)	C16—C17—H17	119.3
C22—O2—H2	109.5	C18—C17—H17	119.3
C1—N1—C2	108.51 (17)	C19—C18—C17	122.0 (2)
C1—N1—C3	113.9 (2)	C19—C18—C13	119.1 (2)
C2—N1—C3	112.13 (18)	C17—C18—C13	118.9 (2)
N1—C1—O1	113.3 (2)	C20—C19—C18	121.2 (2)
N1—C1—H1A	108.9	C20—C19—H19	119.4
O1—C1—H1A	108.9	C18—C19—H19	119.4
N1—C1—H1B	108.9	C19—C20—C11	119.5 (3)
O1—C1—H1B	108.9	C19—C20—H20	120.2
H1A—C1—H1B	107.7	C11—C20—H20	120.2
N1—C2—C12	110.9 (2)	C22—C21—C30	118.9 (2)
N1—C2—H2A	109.5	C22—C21—C3	119.3 (2)
C12—C2—H2A	109.5	C30—C21—C3	121.7 (2)
N1—C2—H2B	109.5	O2—C22—C21	122.8 (2)
C12—C2—H2B	109.5	O2—C22—C23	115.6 (2)
H2A—C2—H2B	108.1	C21—C22—C23	121.6 (2)
N1—C3—C21	111.6 (2)	C24—C23—C22	120.1 (2)
N1—C3—H3A	109.3	C24—C23—H23	119.9
C21—C3—H3A	109.3	C22—C23—H23	119.9
N1—C3—H3B	109.3	C23—C24—C25	121.2 (2)
C21—C3—H3B	109.3	C23—C24—H24	119.4
H3A—C3—H3B	108.0	C25—C24—H24	119.4
C12—C11—O1	123.0 (2)	C26—C25—C24	121.7 (2)
C12—C11—C20	121.7 (2)	C26—C25—C30	119.5 (2)
O1—C11—C20	115.2 (2)	C24—C25—C30	118.9 (2)
C11—C12—C13	119.45 (19)	C27—C26—C25	121.5 (2)
C11—C12—C2	120.0 (2)	C27—C26—H26	119.3
C13—C12—C2	120.5 (2)	C25—C26—H26	119.3
C14—C13—C12	123.26 (18)	C26—C27—C28	119.5 (2)
C14—C13—C18	117.8 (2)	C26—C27—H27	120.3
C12—C13—C18	119.0 (2)	C28—C27—H27	120.3
C15—C14—C13	121.6 (2)	C29—C28—C27	120.8 (2)
C15—C14—H14	119.2	C29—C28—H28	119.6
C13—C14—H14	119.2	C27—C28—H28	119.6
C14—C15—C16	120.7 (3)	C28—C29—C30	121.6 (2)
C14—C15—H15	119.7	C28—C29—H29	119.2
C16—C15—H15	119.7	C30—C29—H29	119.2
C17—C16—C15	119.6 (2)	C29—C30—C25	117.2 (2)
C17—C16—H16	120.2	C29—C30—C21	123.63 (19)
C15—C16—H16	120.2	C25—C30—C21	119.2 (2)
C16—C17—C18	121.4 (2)		
			
C2—N1—C1—O1	64.2 (3)	C13—C18—C19—C20	0.0 (4)
C3—N1—C1—O1	−61.4 (3)	C18—C19—C20—C11	0.6 (4)
C11—O1—C1—N1	−45.3 (3)	C12—C11—C20—C19	−1.3 (4)
C1—N1—C2—C12	−48.9 (3)	O1—C11—C20—C19	−178.8 (2)
C3—N1—C2—C12	77.7 (2)	N1—C3—C21—C22	40.2 (3)
C1—N1—C3—C21	−165.05 (18)	N1—C3—C21—C30	−142.0 (2)
C2—N1—C3—C21	71.3 (2)	C30—C21—C22—O2	178.02 (19)
C1—O1—C11—C12	12.4 (3)	C3—C21—C22—O2	−4.1 (3)
C1—O1—C11—C20	−170.1 (2)	C30—C21—C22—C23	−3.5 (3)
O1—C11—C12—C13	178.6 (2)	C3—C21—C22—C23	174.4 (2)
C20—C11—C12—C13	1.3 (3)	O2—C22—C23—C24	−178.6 (2)
O1—C11—C12—C2	−0.5 (3)	C21—C22—C23—C24	2.8 (4)
C20—C11—C12—C2	−177.8 (2)	C22—C23—C24—C25	0.4 (4)
N1—C2—C12—C11	19.3 (3)	C23—C24—C25—C26	176.7 (2)
N1—C2—C12—C13	−159.8 (2)	C23—C24—C25—C30	−2.7 (3)
C11—C12—C13—C14	−179.3 (2)	C24—C25—C26—C27	−178.9 (2)
C2—C12—C13—C14	−0.3 (3)	C30—C25—C26—C27	0.5 (3)
C11—C12—C13—C18	−0.6 (3)	C25—C26—C27—C28	−1.9 (4)
C2—C12—C13—C18	178.4 (2)	C26—C27—C28—C29	1.1 (4)
C12—C13—C14—C15	177.5 (2)	C27—C28—C29—C30	1.1 (4)
C18—C13—C14—C15	−1.2 (3)	C28—C29—C30—C25	−2.4 (3)
C13—C14—C15—C16	1.3 (4)	C28—C29—C30—C21	176.7 (2)
C14—C15—C16—C17	0.0 (4)	C26—C25—C30—C29	1.6 (3)
C15—C16—C17—C18	−1.2 (4)	C24—C25—C30—C29	−179.0 (2)
C16—C17—C18—C19	−177.5 (2)	C26—C25—C30—C21	−177.5 (2)
C16—C17—C18—C13	1.2 (4)	C24—C25—C30—C21	1.9 (3)
C14—C13—C18—C19	178.7 (2)	C22—C21—C30—C29	−177.9 (2)
C12—C13—C18—C19	0.0 (3)	C3—C21—C30—C29	4.3 (3)
C14—C13—C18—C17	0.0 (3)	C22—C21—C30—C25	1.1 (3)
C12—C13—C18—C17	−178.8 (2)	C3—C21—C30—C25	−176.70 (19)
C17—C18—C19—C20	178.7 (2)		

### Hydrogen-bond geometry (Å, º)

Cg2, Cg3 and Cg5 are the centroids of the C11–C13/C18–C20, C13–C18 and C25–C30 rings, respectively.

**Table:**

*D*—H···*A*	*D*—H	H···*A*	*D*···*A*	*D*—H···*A*
O2—H2···N1	0.84	1.88	2.627 (2)	147
C1—H1*A*···*Cg*3^i^	0.99	2.53	3.501 (3)	169
C2—H2*B*···*Cg*2^i^	0.99	2.86	3.743 (3)	149
C14—H14···*Cg*5^ii^	0.99	2.87	3.723 (3)	150

Symmetry codes: (i) −*x*+1, −*y*+1, −*z*+1; (ii) −*x*+1/2, *y*−1/2, −*z*+3/2.

## References

[bb1] Burke, W. J. (1949). *J. Am. Chem. Soc.* **71**, 609–612.

[bb2] Burke, W. J., Kolbezen, M. J. & Stephens, C. W. (1952). *J. Am. Chem. Soc.* **74**, 3601–3605.

[bb3] Groom, C. R. & Allen, F. H. (2014). *Angew. Chem. Int. Ed.* **53**, 662–671. 10.1002/anie.201306438 24382699

[bb4] Ishida, H. & Sanders, D. P. (2001). *Polymer*, **42**, 3115–3125.

[bb5] Liu, Q.-W., Zhang, M.-J., Wang, X.-Q. & Ma, P.-G. (2005). *Acta Cryst.* E**61**, o4285–o4286.

[bb6] Ma, S.-S., Zhang, M.-J., Yuan, D.-Y. & Qi, Z.-B. (2005). *Acta Cryst.* E**61**, o1370–o1371.

[bb7] Mathew, B. P., Kumar, A., Sharma, S., Shukla, P. K. & Nath, M. (2010). *Eur. J. Med. Chem.* **45**, 1502–1507. 10.1016/j.ejmech.2009.12.058 20116901

[bb8] Rivera, A., Ríos, J., Quevedo, R. & Joseph-Nathan, P. (2005). *Rev. Colomb. Quim* **34**, 105–115.

[bb9] Sarojini, B. K., Narayana, B., Mayekar, A. N., Yathirajan, H. S. & Bolte, M. (2007). *Acta Cryst.* E**63**, o4739.

[bb10] Sheldrick, G. M. (2008). *Acta Cryst.* A**64**, 112–122. 10.1107/S0108767307043930 18156677

[bb11] Sheldrick, G. M. (2015). *Acta Cryst.* C**71**, 3–8.

[bb12] Spek, A. L. (2009). *Acta Cryst.* D**65**, 148–155. 10.1107/S090744490804362X PMC2631630 19171970

[bb13] Stoe & Cie (2001). *X-AREA*. Stoe & Cie, Darmstadt, Germany.

[bb14] Uyar, T., Koyuncu, Z., Ishida, H. & Hacaloglu, J. (2008). *Polym. Degrad. Stab.* **93**, 2096–2103.

[bb15] Yildirim, A., Kiskan, B., Demirel, A. L. & Yagci, Y. (2006). *Eur. Polym. J.* **42**, 3006–3014.

